# High-Throughput Analysis and Characterization of *Astragalus membranaceus* Transcriptome Using 454 GS FLX

**DOI:** 10.1371/journal.pone.0095831

**Published:** 2014-05-14

**Authors:** Xiu-Bo Liu, Ling Ma, Ai-Hua Zhang, Yan-He Zhang, Jing Jiang, Wei Ma, Lei-Ming Zhang, Wei-Chao Ren, Xiang-Jun Kong

**Affiliations:** 1 Heilongjiang University of Chinese Medicine, Harbin, China; 2 Key Laboratory of Forest Tree Genetic Improvement and Biotechnology, Ministry of Education, Northeast Forestry University, Harbin, PR China; 3 Northeast Forestry University, Harbin, PR China; 4 First Affiliated Hospital of Qiqihar Medical University, Qiqihar, PR China; Institute of Biomedical Sciences, Taiwan

## Abstract

*Astragalus membranaceus* (Fisch.) Bge (AR), one of the most important medicinal plants in Asia, was found to exhibit various bioactivities. Due to limited genomic and transcriptomic data, the biosynthetic pathway of the major bioactive compound in AR, is currently unclear. In this study, 454 GS FLX technology was employed to produce a substantial expressed sequence tag (EST) dataset from the AR. In all, 742721 high-quality reads from the AR were produced using Roche GS FLX Titanium. A total of 9893 unique sequences were obtained and annotated by a similarity search against the public databases, and involved in the secondary metabolic pathway, which would facilitate deciphering the molecular mechanism of secondary metabolism in AR. The assembled sequences were annotated with gene names and Gene Ontology (GO) terms. GO revealed the unique sequences that could be assigned to 34 vocabularies. In the KEGG mapping, unique sequences were established as associated with 46 biochemical pathways. These results provided the largest EST collections in AR and will contribute to biosynthetic and biochemical studies that lead to drug improvement. With respect to the genes related to metabolism and biosynthesis pathway were also found. Our work demonstrated the utility of 454 GS FLX as a method for the rapid and cost-effective identification of AR transcriptome, and this EST dataset will be a powerful resource for further studies such as taxonomy, molecular breeding, and secondary metabolism in AR.

## Introduction

Identification of candidate genes involved in the biosynthetic pathway will significantly contribute to the understanding of the biosynthetic and medicinal chemistry of the plant [1]. The sequencing and analysis of expressed sequence tags (EST) has been a primary tool for the discovery of novel genes in plants, especially in plants for which full genome sequences are not currently available [2]–[4]. EST sequencing represents a rapid and cost-effective method for analyzing the transcriptome. Transcriptome sequencing would provide a foundation for detailed studies of gene expression and genetic connectivity with respect to plant secondary metabolism. Various techniques such as microarray, serial analysis of gene expression and massively parallel signature sequencing emerged for high throughput gene expression profiling in the past [5], [6]. However, these techniques are time consuming and become expensive, particularly for the analysis at global level. Advent of next-generation sequencing technologies has led to a revolution in genomics and genetics, and provided a cheaper and faster delivery of sequencing information. The 454 pyrosequencing is the most widely used new platform in de novo sequencing and is applied in medicinal plants [7]. The 454 GS FLX sequencing technology has made EST-based resources more readily accessible for plant transcriptomes [8].

*Astragalus membranaceus* (AR) is one of the most important medicinal plants in Asia, and has received much of the world attention in recent years, as listed in the 2010 edition of Pharmacopoeia of the People’s Republic of China [9]–[11]. This herb has been extensively studied and various bioactivities, such as hypotensive, inducing vasodilatation, antioxidative, immunostimulating, antiviral, protecting the myocardium in diabetic nephropathy, promoting the motility of human spermatozoa, reducing the capillary hyperpermeability and alleviating the dyskinesia; enhancing cardiovascular function, antiaging, hepatoprotective effect, inhibiting cyclooxygenase-2, sterol biosynthesis, and antibacterial [12]–[15]. Clinical research shows that AR can improve cardiovascular function, restore and strengthen immune response, and enhance vitality [16]. Despite the medicinal importance of AR, information on its genetic background is scarce and there is little transcriptome research on this plant. Although the various chemical and pharmacological properties of AR have been extensively studied, the biosynthetic pathway of its compounds remains also poorly understood. EST represents a rapid and cost-effective method for analyzing transcribed genome regions, and is also a powerful approach for discovering new genes and analyzing gene expression profiles [17]. Here, we report the generation of a large-scale EST dataset from AR using the cost-effective 454 GS-FLX pyrosequencing technology. EST dataset can lay a foundation and facilitate AR breeding and taxonomy, and decode secondary metabolism in AR. Transcriptomes and metabolic pathways were analyzed to evaluate the active medicinal compounds in AR and hope that this approach can be used for a variety of medicinal herbs in the future.

## Materials and Methods

### Plant Material

Samples of AR (age 35 days, height 15 cm, seven true leaves) were collected from the Medicinal Botanical Garden of Heilongjiang University of Chinese Medicine. After cleaning, AR leaves were immediately frozen in liquid nitrogen and stored at −80°C until use. AR was dried in 105°C for 0.5 h and 80°C drying to constant weight, vacuum packed until DNA extraction. Voucher specimens for every sampled population were deposited at the College of Pharmacy of Heilongjiang University of Chinese Medicine.

### RNA Extraction, cDNA Library Construction

The total RNA was extracted as previously described [18]. Approximately 2.0 g poly(A)+RNA was isolated using Oligotex, Oligotex mRNA Mini Kit, while cDNA was synthesized using the DNA Synthesis system (Roche). First-strand cDNA synthesis was performed with 3 SMART CDS Primer II A, as described in the provided protocol, using 0.9 g purified poly(A)+RNA (Clontech). Double-stranded cDNA was prepared from 2 L of the first strand by polymerase chain reaction (PCR) with 5 PCR Primer II A in a 100 L reaction and amplified using PCR Advantage II polymerase. The following thermal profile was applied: 1 min at 95°C followed by 30 cycles of 95°C for 15 s, 65°C for 30 s, and 72°C for 2 min (Clontech). Amplified cDNA product was purified with the PureLink PCR Purification Kit (Invitrogen). Three biological replicates were performed for each gene. The resulting cDNA was fragmented and subjected to sequencing on a 454 GS FLX Titanium platform (454 Life Sciences, Roche).

### Sequence Assembly

Sequencing was based on the 454 GS FLX platform and Titanium reagents. Preparation of the 454 library was performed according to the supplier’s instructions. Approximately 5 µg of amplified cDNA was nebulized and selected for length, which ranged from 300 to 800 bp. The DNA fragments were then denatured to generate single-stranded DNA, which was then amplified by emulsion PCR for sequencing. The sequencing of the libraries was performed on a 454 GS FLX.

### Unigene Function Annotation, GO Classification, and Metabolic Pathway Analysis

Unigene sequences were searched against Gene Ontology (GO; www.geneontology. org), NCBI’s nr (http://www.ncbi.nlm.nih.gov/), KEGG (www.genome.jp/kegg) and Orthologous Groups of proteins (www.ncbi.nlm.nih.gov/COG) protein databases using BLASTX to identify the proteins that had the highest sequence similarity with the given unigenes along and to retrieve their functional annotations. GO is a standardized gene functional classification system that offers a dynamic-updated controlled vocabulary and a strictly defined concept to comprehensively describe properties of genes and their products in any organism. It has three ontologies: molecular function, cellular component and biological process. The Blast2GO program was used to assign GO annotations to the unigenes. The KEGG database contains metabolic pathways that represent molecular interactions and reaction networks. We used the KEGG annotation to assign pathway annotations to the unigenes that were assigned to special biochemical pathways according to the KEGG standards using BLASTX. The COG is a database in which orthologous gene products are classified. The unigenes were aligned to the COG database to predict and classify the possible functions of the unigenes.

## Results and Discussion

### Sequence Assembly

The dataset of raw reads was deposited in NCBI database under accession number and contains about 742721 reads 50 bp in length totalling 306805437 bp. Reads shorter than 30 bp after pre-processing were excluded. After removing redundant reads, filtering reads of low quality, trimming low-quality ends and removing adapters, the size of the dataset was about 306 Mbp. Using a series of normalization, correction and quality-filtering algorithms, the 454 sequencing data were processed to screen and filter for weak signals and low-quality reads, and to trim the read ends for low quality sequences. All processing and analyses of the sequencing data was performed with the GS-FLX Software (454 Life Sciences, Roche).

### Sequence Generation

**Figure 1:**
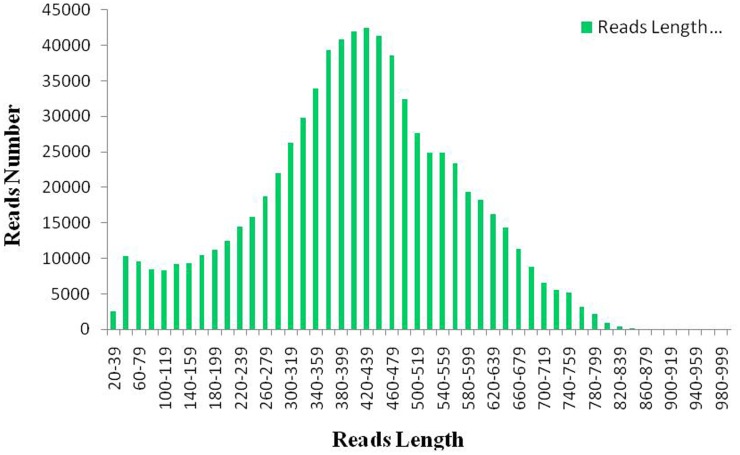
Sequence length distribution of *AR* 454 ESTs and GenBank ESTs. Y-axis: count number; X-axis: size in bp.

In order to distinguish redundant sequences from homologous sequences, “unigene” was used in this study to minimize redundancy, each unique sequence was assigned a unigene ID according to the accession number of the best-hit homologue in the Nr database, and finally 9732 unigenes were obtained. Figure 1 illustrates the sequence length distributions of the ESTs derived from 454 sequencing and Gen-Bank. A half plate 427 run yielded 55.9 MB bases from 639061 reads with an average 1069.5 bp. The sequences were deposited at NCBI under the accession number. After trimming the adapter sequences and removing low quality sequences, a total of 306805437 clean reads were assembled. Assembly of the trimmed, size-selected ESTs produced 15167 contigs with a mean length of 1069.5 bp, as well as an additional 84393 singletons, for a total of 9893 unigenes (contigs and singletons). Finally, 9893 unigenes were identified from contigs dataset. The largest unigene is 7524 bp in length, and the N50 length is 1205 bp. To date, only a small number of AR ESTs have been deposited in GenBank, alignment analysis showed that the majority of ESTs can be found in our 454 sequence collection.

### Gene Ontology Annotation

**Figure 2:**
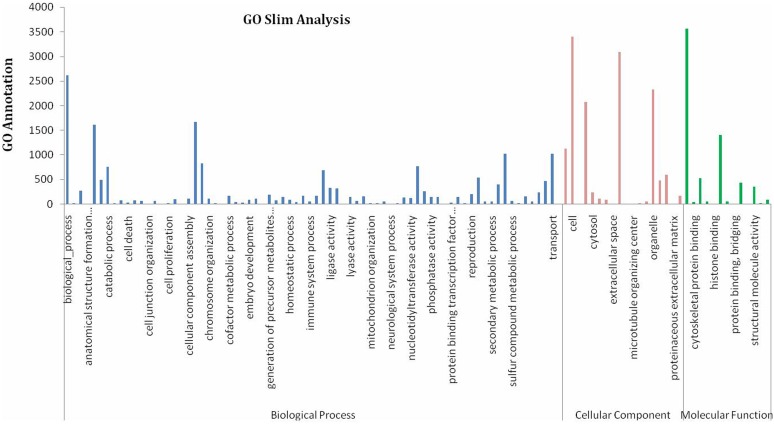
Gene ontology classification assigned to the *AR* unigenes. GO categories of biological process, cellular component and molecular function for the transcriptome of AR. Histogram presentation of the gene ontology classification. The results are summarized in the three main GO categories: biological process, cellular component and molecular function. The *x*-axis represents the categories the unigenes belong to and the *y*-axis the unigene numbers.

A further functional classification of all unigenes was performed using a set of plant-specific GO slims (Figure 2). A total of 9732 unigenes (98.37%) were assigned to 34 functional groups using GO assignments, including biochemistry, cell apoptosis, growth, development, and metabolism. Within each of the three main GO categories, biological process (2615 unigenes), cellular component (1129 unigenes) and molecular function (3564 unigenes), the dominant subcategories were ‘‘biological process’’, ‘‘cell assembly’’ and ‘‘cellular binding’’, respectively. ‘‘secondary metabolic process’’, “organelle”, ‘‘extracellular space’’, and ‘‘transport’’ were also well represented. However, few genes were assigned to the category ‘‘cell death’’, and no genes were found in the clusters of ‘‘cell killing’’, ‘‘nervous system procedure’’ or ‘‘cell proliferation’’.

### COG Annotation

**Figure 3:**
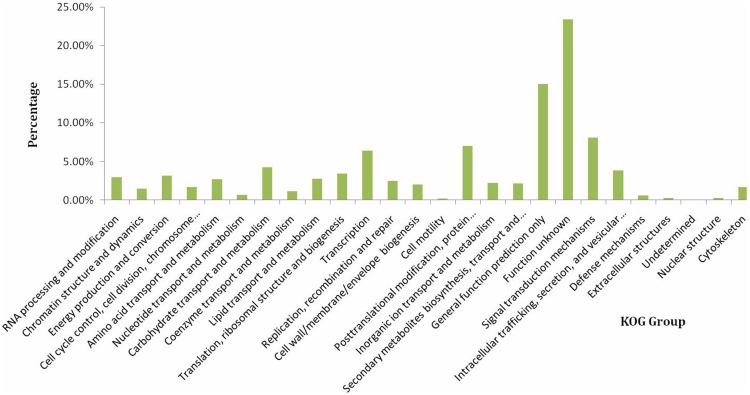
COG classification assigned to the AR unigenes. Histogram presentation of clusters of orthologous groups (COG) classification. Out of 52752 nr hits, 23319 sequences have a COG classification among the 26 categories.

To further evaluate the completeness of our transcriptome library and the effectiveness of our annotation process, we used the annotated unigene sequences to search for the genes in the COG classifications. Among the 26 COG categories (Figure 3) that were assigned to unigenes, the “Function unknown” cluster represented the largest group (5998, 23.40%), the “general function prediction” cluster represented the larger group (846, 15.01%) followed by “signal transduction mechanisms” (2079, 8.11%), “posttranslational modification, protein turnover, chaperones” (1792, 6.99%), “transcription” (1633, 6.37%), “carbohydrate transport and metabolism” (108, 4.23%), “intracellular trafficking, secretion, and vesicular transport” (984, 3.84%), “translation, ribosomal structure and biogenesis” (879, 3.43%), “energy production and conversion” (808, 3.15%), “RNA processing and modification” (757, 2.95%), “lipid transport and metabolism”(711, 2.77%), “amino acid transport and metabolism” (699, 2.73%), “replication, recombination and repair” (635, 2.48%), “inorganic ion transport and metabolism” (572, 2.23%), “secondary metabolites biosynthesis, transport and catabolism” (553, 2.16%), while, cell wall/membrane/envelope biogenesis (520, 2.03%), cell cycle control, cell division, chromosome partitioning (438, 1.71%) and chromatin structure and dynamics (433, 1.69%), coenzyme transport and metabolism (302, 1.18%), nucleotide transport and metabolism (181, 0.71%) and defense mechanisms (156, 0.61%), extracellular structures (66, 0.26%), nuclear structure (61, 0.24%) and cell motility (54, 0.21%) represented the smallest groups.

### Genes Involved in Secondary Metabolism

**Figure 4:**
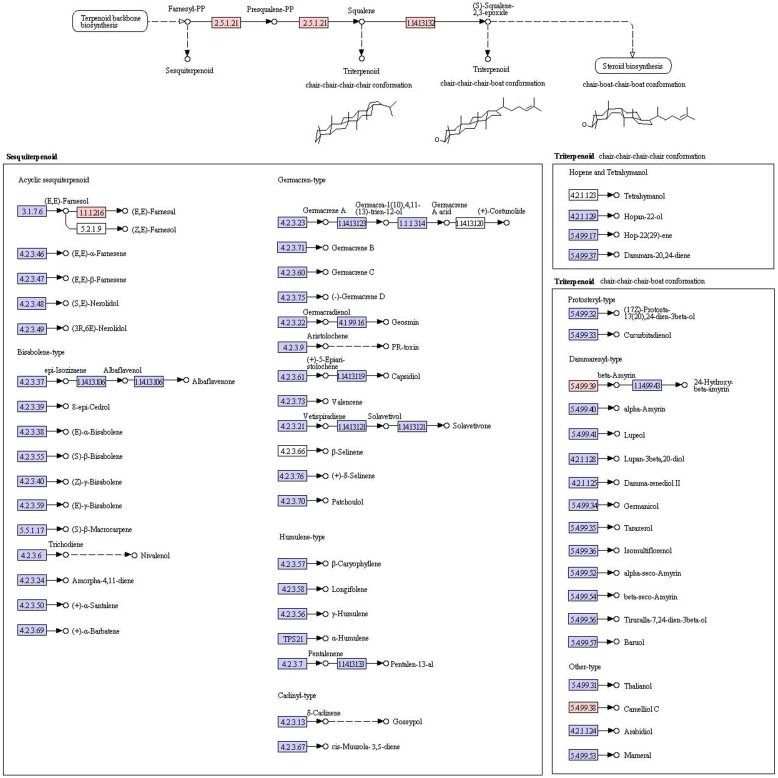
Pathway of Sesquiterpenoid and triterpenoid biosynthesis in the AR.

**Figure 5:**
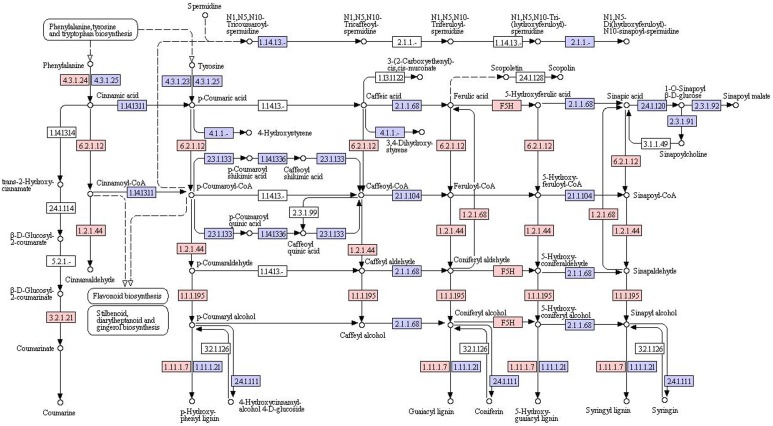
Key enzymes and proteins in regulating biosynthesis of phenolic acids in AR.

**Figure 6:**
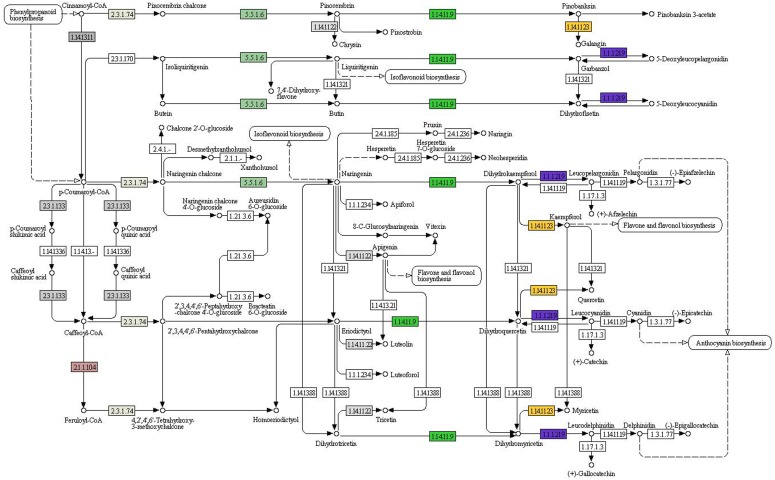
Key enzymes and proteins in regulating biosynthesis of flavonoid biosynthesis in AR.

KEGG analysis provides an alternative functional annotation of genes. We next determined the overall expression levels for each KEGG pathway as described above for GO codes. To identify the biological pathways that were active in *AR*, we mapped the 9893 annotated sequences to the reference canonical pathways in KEGG. A total of 2877 unigene sequences were assigned to 45 KEGG pathways (Table 1). The most pathways were “genetic information processing”, “metabolic pathways”, and “human diseases”, “organismal systems”, “environmental information processing” and “cellular processes”. The metabolism pathways were involved in the glycan biosynthesis and metabolism (36 unigenes), including “N-glycan biosynthesis” (6 unigenes) and “various types of N-glycan biosynthesis” (11 unigenes), “other types of o-glycan biosynthesis” (2 unigenes); “glycosylphosphatidylinositol-anchor biosynthesis” (3 unigenes) and “lipopolysaccharide biosynthesis” (3 unigenes), and “other glycan degradation” (6 unigenes). The pathways with the highest expression levels were ‘metabolism of terpenoids and polyketides’ and ‘biosynthesis of other secondary metabolites’ with mean coverage values of 68 and 90, respectively. The metabolism pathways are involved in the metabolism of terpenoids and polyketides (68 unigenes), including “terpenoid backbone biosynthesis” (21 unigenes) and “sesquiterpenoid and triterpenoid biosynthesis” (11 unigenes) (Figure 4), “diterpenoid biosynthesis” (6 unigenes), “carotenoid biosynthesis” (16 unigenes) and “brassinosteroid biosynthesis” (1 unigene), and “zeatin biosynthesis” (3 unigenes), “tetracycline biosynthesis” (4 unigenes) and “polyketide sugar unit biosynthesis” (1 unigene). Metabolism pathways are involved in the biosynthesis of secondary metabolites (90 unigenes), including “phenylpropanoid biosynthesis” (55 unigenes) (Figure 5) and “flavonoid biosynthesis” (1 unigene) (Figure 6), “anthocyanin biosynthesis” (1 unigene), “isoquinoline alkaloid biosynthesis” (1 unigene) and “tropane, piperidine and pyridine alkaloid biosynthesis” (11 unigenes), and “caffeine metabolism” (1 unigene), “betalain biosynthesis” (2 unigenes) and “streptomycin biosynthesis” (4 unigenes), “novobiocin biosynthesis” (3 unigenes).

**Table 1:** Pathway classification of *Astragalus membranaceus.*

Category	Pathway	Count
Metabolism	Carbohydrate Metabolism	164
	Energy Metabolism	152
	Lipid Metabolism	106
	Nucleotide Metabolism	36
	Amino Acid Metabolism	158
	Metabolism of Other Amino Acids	56
	Glycan Biosynthesis and Metabolism	36
	Metabolism of Cofactors and Vitamins	101
	Metabolism of Terpenoids and Polyketides	68
	Biosynthesis of Other Secondary Metabolites	90
	Xenobiotics Biodegradation and Metabolism	41
Genetic Information Processing	Transcription	99
	Translation	399
	Folding, Sorting and Degradation	259
	Replication and Repair	34
Environmental Information Processing	Membrane Transport	13
	Signal Transduction	137
	Signaling Molecules and Interaction	1
Cellular Processes	Transport and Catabolism	94
	Cell Motility	8
	Cell Growth and Death	33
	Cell Communication	6
Organismal Systems	Immune System	12
	Endocrine System	41
	Digestive System	30
	Excretory System	4
	Nervous System	34
	Environmental Adaptation	85
Human Diseases	Cancers	51
	Immune Diseases	5
	Neurodegenerative Diseases	79
	Substance Dependence	29
	Cardiovascular Diseases	9
	Endocrine and Metabolic Diseases	18
	Infectious Diseases	295

AR belongs to leguminous plant of the Astragalus family, and is one of the most popular traditional Chinese medicines, is widely used as an antidiabetic, immunostimulating, hepatoprotective, cadiotonic, and antiviral drug [19]. It has been widely used in China and East Asia area for many years to treat myocardial ischemia, liver fibrosis, chronic nephritis, diabetes, etc [20]. Phytochemical studies have shown that the main active components in medicinal plants are secondary metabolites which are produced during the growth and development of plants as they adapt to the environment [21], [22]. These components are usually used as markers for identification and quality evaluation of traditional Chinese medicine. To the best of our knowledge, however, the genomics or transcriptome data or the biosynthetic pathway of the major bioactive compound in AR are still not completely elucidated until now.

In this study, we had totally identified 9893 unique sequences which involved in primary and secondary metabolites biosynthesis, earching the annotation information from the KEGG, COG, GO and Nr database. All the genes in these pathways were found in the transcriptome dataset. These unigene sequences and their annotations will provide a valuable resource for investigating specific processes, functions and pathways in *AR*, and will allow for the identification of novel genes involved in the secondary metabolite synthesis pathways. KEGG pathway analysis suggested that transcripts of the glycan biosynthesis, metabolism of terpenoids and polyketides, biosynthesis pathways of other secondary metabolites were highly abundant in *AR*. Sequence similarity searches against public databases identified the unigenes that could be annotated with gene descriptions, conserved protein domains, and/or gene ontology terms [23]. Some of the unigenes were assigned to putative metabolic pathways. Targeted searches using these annotations identified most of the genes that are associated with several primary metabolic pathways and natural product pathways. These genes, such as those that code for glycan biosynthesis, terpenoids and polyketides biosynthesis pathways *etc.*, are important for the quality of *AR* as a medicinal plant. This is the first time that the novel candidate genes of these secondary pathways have been discovered in AR.

The quality of herbal medicine has been very difficult to control and to evaluate, primarily because of the complexity and incomplete knowledge of the active compounds. The primary methods that have been used for quality evaluation of herbal medicines are chemical and pharmacological analyses. However, content and fingerprint analysis of one or more of these compounds are not indicative for the medicinal value of the plant, and our genomic approach provides more comprehensive understanding of AR as medicinal plant. Our study generated gene expression data for terpenoids and fatty acid biosynthesis and increased valuable knowledge on other AR compounds. These results provided a meaningful basis for evaluating the bioactive components and their action mechanisms of AR. In this study, we obtained a sufficient amount of transcriptomic data from AR, and carried out an integrated analysis on the gene expression of active compounds. It can be used to produce genetically improved varieties of *AR* with increased secondary metabolite yields, different compound compositions and better medicinal characteristics.

## Conclusion

The transcriptomes can be informative for the analysis of active medicinal compounds in herbal plants. To explore the transcriptome of *AR* and identify genes involved in the metabolism pathway, the 454 GS FLX technology was used in this study to generate substantial transcriptome sequence data. With this work, we initiated a large-scale investigation of the transcriptome of *AR* in terms of functional genomics, molecular biology and biochemistry. A large number of novel candidate genes involved in primary and secondary metabolites biosynthesis, were identified in our EST dataset. A total of 9732 sequences have been successfully annotated with GO terms based on the known sequences, and unique sequences are involved in the secondary metabolic pathway, which would facilitate deciphering the molecular mechanism of secondary metabolism in AR. Our study provided an initial description of the expression profiles of AR, and also gives a first insight into the gene expression patterns of AR. More importantly, the putative transcriptome information explored in this study will provide a significant contribution towards understanding the metabolism and biosynthesis and may help us to enhance the quality of AR.
